# Health challenges in Africa and the way forward

**DOI:** 10.1186/1755-7682-1-27

**Published:** 2008-12-18

**Authors:** Joses Muthuri Kirigia, Saidou Pathe Barry

**Affiliations:** 1 Division of Health Systems and Services Development, World Health Organization Regional Office for Africa, Brazzaville, Congo

## Abstract

Africa is confronted by a heavy burden of communicable and non-communicable diseases. Cost-effective interventions that can prevent the disease burden exist but coverage is too low due to health systems weaknesses. This editorial reviews the challenges related to leadership and governance; health workforce; medical products, vaccines and technologies; information; financing; and services delivery. It also provides an overview of the orientations provided by the WHO Regional Committee for Africa for overcoming those challenges. It cautions that it might not be possible to adequately implement those orientations without a concerted fight against corruption, sustained domestic and external investment in social sectors, and enabling macroeconomic and political (i.e. internally secure) environment.

## Overview of disease burden

Out of 58.03 million people who died globally in 2005, 10.9 million (18.8%) were from the WHO African Region [1]. Majority of deaths (64%) that occurred in the Region resulted from HIV/AIDS (19%), lower respiratory infections (10%), malaria (8%), diarrhoeal diseases (7%), cerebrovascular disease (4%), ischaemic heart disease (3%), tuberculosis (3%), measles (3%), low birth weight (2%), birth asphyxia and birth trauma (2%) and maternal conditions (2%). Even though effective public health interventions that could have prevented most of deaths exist, coverage is low due to weak and under-resourced health systems. Some of the weaknesses can be attributed to challenges related to leadership and governance; health workforce; medical products, vaccines and technologies; information; financing; and services delivery [2].

## Challenges

Firstly, there are serious leadership and governance challenges that include weak public health leadership and management [3]; inadequate health-related legislations and their enforcement; limited community participation in planning, management and monitoring of health services; weak inter-sectoral action; horizontal and vertical inequities in health systems [4]; inefficiency in resource allocation and use [5]; and weak national health information and research systems [6].

Secondly, extreme shortages of health workers exist in 57 countries of which 36 are in Africa [8]. The crisis has been exacerbated by inequities in workforce distribution and brain drain. Thus, the delivery of effective public health interventions to people in need is compromised particularly in remote rural areas.

Thirdly, there is rampant corruption in medical products and technologies procurement systems, unreliable supply systems, unaffordable prices, irrational use, wide variance in quality and safety [2]. This has contributed to current situation where 50% the population in the Region lack of access to essential medicines [6].

Fourthly, there is a dearth of information and communications technology (ICT) and mass Internet connectivity, compounded by a paucity of ICT-related knowledge and skills limiting capacities of national health management information systems (HMIS) to generate, analyze and disseminate information for use in decision-making [7].

Fifthly, health financing in the Region is characterized by low investment in health, lack of comprehensive health financing policies and strategic plans, extensive out-of-pocket payments, lack of social safety nets to protect the poor, weak financial management, inefficient resource use, and weak mechanisms for coordinating partner support [9].

Finally, in terms of service delivery, the lack of effective organisation and management of health services combined with the above indicated challenges have in tandem led to the current situation where 47% of the population have no access to quality health services, 59% of pregnant women deliver babies without the assistance of skilled health personnel. In relation to water and sanitation which contribute to reducing burden of communicable diseases, 64% of the population lack sustainable access to improved sanitation facilities, and 42% lack sustainable access to an improved water source [6].

## The way forward

### Orientations for strengthening national health systems

Cognizant of the abovementioned challenges, the 46 Ministers of Health from the African Region adopted and signed the Ouagadougou Declaration [10] that proposes ways of addressing health system challenges. The Ouagadougou Declaration urges Member States to update their national health policies and plans according to the primary health care (PHC) approach; promote inter-sectoral collaboration and public-private partnership to address broad determinants of health; improve health workforce production and retention; set up mechanisms for increasing availability and accessibility of essential medicines, health technologies and infrastructure; strengthen health information systems; develop and implement strategic health financing policies and plans; promote health awareness and build behavioural change capacities among communities.

Subsequently, the fifty-eighth WHO Regional Committee for Africa endorsed a framework for implementing the Ouagadougou Declaration [11]. The framework proposes to countries generic interventions for addressing the health systems challenges.

Firstly, regarding leadership and governance, it proposes updating of national health policy and strategic plan; updating and enforcing public health laws; and strengthening mechanisms for transparency and accountability and intersectoral collaboration [10].

Secondly, concerning HRH, it recommends generation and use of evidence in HRH policy development, planning and management; reinforcing health training institutions capacity; strengthening HRH leadership and management capacities; implementing HRH retention strategies; and increasing fiscal space for HRH development [10].

Thirdly, with regard to medical products and technologies, it proposes development of formulae for determining the requirements and forecasting for medicines, commodities, essential technologies and infrastructure; and creation of a transparent and accountable procurement system [10].

Fourthly, relating to health information and research, the Declaration on Primary Health Care and Health Systems suggests development and implementation of a comprehensive HMIS policy and strategic plan; and establishment of a functional national HMIS by leveraging ICT [10]. In addition, the Algiers Declaration [12] recommends development and retention of a critical mass of human resources for health research; updating of national health research policies and strategic frameworks; strengthening of south-south and north-south research cooperation; establishment of mechanisms for scientific and ethical oversight of research; acquisition of ICT for research and information; and allocation of at least 2% of national health expenditure and at least 5% of external aid for health to research.

Fifthly, pertaining to health financing, it recommends development of a comprehensive health financing policy and a strategic plan; institutionalization of national health accounts and efficiency monitoring; strengthening of financial management skills at all levels; allocation of at least 15% of the national budget to health development; implementation of the Paris Declaration on aid effectiveness; and develop social protection mechanisms [10].

Finally, as regards service delivery, the Declaration proposes building on the elements of essential health services, mode of delivery and costs; development of norms, standards and procedures for service provision and health infrastructure construction and maintenance; formulation of integrated service delivery model at all levels including the referral system; development of mechanisms to involve all private health providers in provision of essential health services; development and implementation of multi-sectoral health promotion policies and strategies to optimize community involvement in health development [10].

## Conclusion

Effective public health interventions are available to curb the heavy disease burden in Africa. Unfortunately, health systems are too weak to efficiently and equitably deliver those interventions to people who need them, when and where needed. Fortunately, the health policy-makers know what actions ought to be implemented to strengthen health systems. However, it might not be possible to adequately implement those actions without a concerted and coordinated fight against corruption, sustained domestic and external investment in social sectors (e.g. health, education, water, sanitation), and enabling macroeconomic and political (i.e. internally secure) environment.

## Abbreviations

HMIS: Health management information systems; HRH: Human resources for health; ICT: Information and communications technology; PHC: Primary health care; WHO: World Health Organization

## Competing interests

The authors declare that they have no competing interests.

## Authors' contributions

JMK and SPB contributed equally in drafting the editorial. All authors read and approved the final manuscript.
